# Chloroform exposure in air and water in Swedish indoor swimming pools—urine as a biomarker of occupational exposure

**DOI:** 10.1093/annweh/wxad035

**Published:** 2023-06-20

**Authors:** Oskar Ragnebro, Kristin Helmersmo, Louise Fornander, Raymond Olsen, Ing-Liss Bryngelsson, Pål Graff, Jessica Westerlund

**Affiliations:** School of Medical Sciences, Örebro University, 701 82 Örebro, Sweden; National Institute of Occupational Health (STAMI), 0363 Oslo, Norway; Department of Occupational and Environmental Medicine, Faculty of Medicine and Health, Örebro University, 701 82 Örebro, Sweden; National Institute of Occupational Health (STAMI), 0363 Oslo, Norway; Department of Occupational and Environmental Medicine, Faculty of Medicine and Health, Örebro University, 701 82 Örebro, Sweden; National Institute of Occupational Health (STAMI), 0363 Oslo, Norway; Department of Occupational and Environmental Medicine, Faculty of Medicine and Health, Örebro University, 701 82 Örebro, Sweden

**Keywords:** chloroform, disinfection by-products, exposure assessment, swimming pools, trihalomethanes, urine

## Abstract

**Introduction:**

Disinfection by-products are produced in water disinfected with chlorine-based products. One such group is trihalomethanes, and chloroform is the most abundant trihalomethane in swimming pool areas. Chloroform can be absorbed by inhalation, ingestion, and dermal absorption, and is classified as possibly carcinogenic.

**Aim:**

To investigate if chloroform concentrations in air and water affect the chloroform concentration in urine samples of exposed swimming pool workers.

**Methods:**

Workers from 5 adventure indoor swimming pools carried personal chloroform air samplers and provided up to 4 urine samples during one workday. Chloroform concentrations were analyzed with a linear mixed model analysis to investigate a possible correlation between air and urine concentrations.

**Results:**

The geometric mean chloroform concentration was 11 μg/m^3^ in air and 0.009 µg/g creatinine in urine among individuals with ≤2 h at work, 0.023 µg/g creatinine among those with >2–5 working hours, and 0.026 µg/g creatinine in the group with >5–10 working hours. A risk of higher levels of chloroform in urine was associated with longer hours at work (≤2 h versus >5–10 h, odds ratio [OR] 2.04, 95% confidence interval [CI] 1.25–3.34), personal chloroform concentrations in air (≤17.00 µg/m^3^ versus >28.00 µg/m^3^, OR 9.23, 95% CI 3.68–23.13) and working at least half the working day near the swimming pools (OR 3.16, 95% CI 1.33–7.55). Executing work tasks in the swimming pool water was not associated with higher chloroform concentrations in urine compared to only working on land (OR 0.82, 95% CI 0.27–2.45).

**Conclusion:**

There is an accumulation of chloroform concentrations in urine during a workday and a correlation between personal air and urine concentrations of chloroform among workers in Swedish indoor swimming pools.

**What’s important about this paper?**

Chloroform exposures among workers in swimming facilities are frequently assessed using stationary sampling, which does not reflect exposure variability among people. This study measured chloroform exposure using personal air sampling and urine sampling and found a statistically significant relationship between chloroform concentrations in air and in urine. This suggests chloroform in urine may be a useful exposure metric for workers in swimming facilities.

## Introduction

Swimming pools are a common sight around the world today, and there are approximately 450 public indoor swimming pools in Sweden. Indoor swimming pools are used for both recreational and health beneficial purposes, such as rehabilitation and exercise. However, health hazards such as accidents and infectious diseases are also associated with the use of swimming pools. As many people use the same pools, the risk of spreading infectious diseases increases. To keep the facilities safe for staff and visitors, the water must be disinfected (WHO 2006). The most common disinfectants for Swedish indoor swimming pools are based on chlorine, such as sodium and calcium hypochlorite (Eriksson et al. 2015), which keeps the facilities safe from most infections if recommendations are followed (WHO 2006). With the decreased risk of infectious diseases, the research on health issues associated with swimming pools has focused on the chemical hazards of disinfection. The disinfection of the pool water is a complicated task, as the need for disinfection depends on variables such as water source, choice of disinfection method, and the number of visitors.

Disinfection of swimming pool water with chlorine is associated with the production of different unwanted by-products called disinfection by-products (DBPs). DBPs are created from reactions with organic and inorganic matter from the source water and swimmers (Lahl et al. 1981; Kanan and Karanfil 2011). Organic matter from swimmers originates from their bodies, like sweat, urine, and saliva, as well as from personal care products, such as lotion or cosmetics. The quantity and variation of DBPs produced depend on factors such as inorganic and organic matter load and variation in chlorination levels (Kim et al. 2002). Many of these DBPs have documented health risks. Trihalomethanes (THMs) are a group of organic DBPs, some of which have been shown to have carcinogenic effects (Simard et al. 2013). The most abundant THM found in swimming pool air is chloroform (Westerlund et al. 2015). THMs are formed in swimming pool water but due to their volatility they are also found in swimming pool air (Lahl et al. 1981).

The Swedish Work Environment Authority (SWEA) has set regulations for air concentrations of pollutants occurring in Swedish workplaces. These regulations set an upper limit for chloroform in the breathing zone air of 10,000 µg/m^3^ (Swedish Work Environment Authority 2018). An earlier study of Swedish indoor swimming pools that measured chloroform concentration in the breathing zone air around workers found a mean of 30 μg/m^3^ (Westerlund et al. 2015). Further, studies from Spain and Italy have measured ambient air concentrations of 21–340 μg/m^3^ at indoor swimming pools (Aggazzotti et al. 1998; Fantuzzi et al. 2001; Caro and Gallego 2008; Aprea et al. 2010). Concentrations of THMs in swimming pool water are regulated by the Swedish Public Health Agency’s guidelines for swimming pools, with an upper limit of 100 µg/L (Public Health Agency of Sweden 2021).

Chloroform is classified as a possibly carcinogenic substance to humans (Group 2B) by the International Agency for Research on Cancer (IARC) (IARC 1999). Animal studies have shown the carcinogenic effects of THMs in rats, in which increased concentrations of chloroform in ambient air and drinking water were associated with an increased risk of renal and liver tumors (Richardson et al. 2007). A correlation between long-term exposure to THMs and bladder cancer has also been seen in humans (Costet et al. 2011), and an epidemiological study from Spain showed an increased risk of bladder cancer with more swimming pool exposure (Villanueva et al. 2007), indicating that even lower exposure levels than set regulations could have long-term carcinogenic effects.

THMs can enter the body through different pathways: ingestion, inhalation, and dermal absorption. In a swimming pool situation, the primary pathways are inhalation from the surrounding air and dermal absorption from being in the pool water. The major pathway of THM uptake in a swimming pool environment is inhalation. This was concluded by having test subjects use scuba equipment and therefore eliminating the inhalation uptake while in the pool. This showed a dermal uptake of THMs among the subjects in scuba equipment compared to other subject who was on dry land during the study, from which the conclusion could be made that THMs enter the body by both dermal absorption and inhalation, though predominantly by inhalation (Erdinger et al. 2004). Chloroform is mainly eliminated from the human body by expired air, as carbon dioxide or unchanged, but also via urine and feces (Agency for Toxic Substances and Disease Registry 1997).

Earlier studies on swimmers and workers have shown an association between exposure to indoor swimming pools and increased concentrations of THMs in urine, blood and exhaled breath (Cammann and Hubner 1995; Caro et al. 2007; Font-Ribera et al. 2016, 2019). The increased concentrations are quickly lowered after leaving the environment and levels are back to normal within 15 h (Cammann and Hubner 1995; Caro et al. 2007). One of the studies also show that elevated levels of THMs can be seen in blood samples within minutes of exposure (Cammann and Hubner 1995).

The aim of this study was to investigate if chloroform concentrations in air and water affect the chloroform concentration in urine samples of exposed swimming pool workers.

## Methods

### Study design

This study is a cross-sectional study completed in the years 2019 and 2020 in Sweden. This design was chosen in order to study the correlation of exposure to airborne chloroform and chloroform in water with concentrations of chloroform in urine samples among indoor swimming pool workers in adventure swimming pool facilities.

### Study objects and subjects

Five swimming pool facilities in Sweden were contacted and agreed to participate in the study. They were located in the counties of Örebro, Dalarna, Sörmland, and Östergötland. Data collection was completed during one day at four of the locations and two days in one of the facilities to enable the participation of more staff. The facilities varied in size, with between 10 to 32 employees and 400 to 2,500 visitors per day. All locations had an indoor water park profile, with both regular indoor swimming pools and adventure-themed pools with more water motion and higher water temperatures. To be included in the study, the subjects must not have worked in the previous two days and needed to work more than 4 h during the day of testing. At these five locations, 41 workers met the inclusion criteria, and all 41 agreed to be a part of the study via an informed consent. The employees had varying work tasks during the day of the investigation, performing administrative work tasks outside the pool area as well as lifeguard duties inside the pool area. Some of them were also swimming school instructors who taught in the swimming pool water. During the day of the investigation, the participants recorded their work tasks in a work diary. Height and weight were recorded to calculate body mass index (BMI), and the subjects filled out a form about gender, age, smoking habits, and years employed at the workplace. Ethical approval for this project was obtained from the Swedish Ethical Review Authority in Uppsala, reference number 2018-314.

### Sampling

Sampling of air, urine, and swimming pool water to determine chloroform concentrations was done during the day of the study at each location. The included study participants carried a chloroform air sampler within their breathing zone for the whole work shift. If the participants executed work tasks in the swimming pool water, their sampling equipment was placed on land in order to avoid water contamination. Air samples were collected with a method based on EPA method TO-17 (US Environmental Protection Agency 1999) and developed at the Department of Occupational and Environmental Medicine at Örebro University Hospital. Chloroform was collected by Universal Mix multi-bed sorbent tubes containing the adsorbents Tenax TA, Carbograph 1TD, and Carboxen 1003 (Markes International, Inc., Sacramento, CA, USA). Sampling was done by connecting the sampling tube to an AirChek XR5000 air sampling pump (SKC, Inc., Pittsburgh, PA, USA) operating at an airflow of 0.01 L per minute and placing the tube within the breathing zone of the subject. There was a maximum sampling time of four hours per testing tube; for longer testing periods, the tubes were replaced after 4 h to prevent an overload.

The subjects were also asked to provide up to four urine samples during the test period. The swimming pool water was also sampled in the different swimming pools at each facility. The urine and water samples were collected in 100 mL cups with screw caps (Sarstedt AG & Co, Nümbrecht, Germany). After a sample was collected, 10 mL of urine or water were pipetted (Thermo Fisher Scientific Inc., Waltham, MA, USA) into a 20 mL screw cap headspace vial (VWR, Radnor, PA, USA) prefilled with 0.52 g of sodium chloride. They were then stored in an Asaklitt portable refrigerator (Clas Ohlson AB, Insjön, Sweden) with a temperature range between +2 °C and +8 °C until they reached the freezer at the laboratory, which had a temperature of −19 °C.

### Analysis

Analysis of chloroform in air was based on EPA method TO-17 (US Environmental Protection Agency 1999). Prior to analysis, a dry purge was performed on all air samples to remove excess water from sorbent tubes. The dry-purge was executed at a helium gas flow of 50 mL/min on a TC-20 sample conditioning and dry-purge rig (Markes International, Inc., Sacramento, CA, USA). The air samples were then analyzed for chloroform using automated thermal desorption (ATD TD 100; Markes International, Inc., Sacramento, CA, USA), gas chromatography (GC 7890B; Agilent, Santa Clara, CA, USA) with a Rxi-5Sil-MS column (60 m × 0.25 mm × 1.0 µm, Restek, Bellefonte, PA, USA) and a mass spectrometry detection (MS 5977; Agilent, Santa Clara, CA, USA). Helium was used as a carrier gas. The sampled tubes were desorbed for 5 min at 250 °C with a helium gas flow of 50 mL/min and cryofocused at 5 °C on a cold trap containing Tenax TA. The sample was then split injected (outlet split 10 mL/min, desorb flow 50 mL/min) onto the GC by heating the cold trap to 250 °C for 4 min. Details of the ATD and GC method are described in Supplementary Material 1. Detection of chloroform was made by acquisition in single ion monitoring (SIM) mode (m/z 83 and 85). The chromatograms were integrated and evaluated using a computer software (G1701DA MSD ChemStation Version D.00.00.38, Agilent tech, USA). Chloroform standards (0.75–60 mg/L) were prepared with chloroform (83626.290-1L; VWR Chemicals, Leuven, Belgium) and methanol (83626-1L; Sigma Aldrich, Damstadt, Germany). The method was linear between 0.75–1000 mg/L and the limit of quantification (LOQ) for chloroform was a signal to noise ratio (S/N) larger than 10. The lowest concentration for chloroform was 0.75 ng per sample, well above the LOQ. A flow rate of 0.01 L per minute and a sampling time of four hours resulted in a corresponding air concentration of 0.31 μg/m^3^. The relative standard deviation (SD) was 30% at 0.75 mg/L and below 15% for the calibration standards 5.0–60 mg/L. Chloroform in air samples was analyzed at the Department of Occupational and Environmental Medicine at Örebro University Hospital, Örebro, Sweden.

To determine the concentration of chloroform in the urine and water samples, they were analyzed with an automatic headspace in-tube extraction (ITEX) method (autosampler PAL RSI 85; CTC Analytics, Zwingen, Switzerland) coupled to a gas chromatograph (GC 6890; Agilent Technologies, Santa Clara, CA, USA) equipped with a RTX-VMS pre-column (2.5 m, Restek, Bellefonte, PA, USA) and a 19920 column (60 m × 0.32 mm × 1.8 µm, Restek, Bellefonte, PA, USA) and a mass spectrometry detector (MS 5973; Agilent Technologies, Santa Clara, CA, USA). Helium 6.0 was used as a carrier gas. A splitless injection was used with a temperature of 170 °C and a solvent delay of 10 min. Details of the ITEX and GC method are described in Supplementary Material 2. The identification of chloroform was made by acquisition in single ion monitoring (SIM) mode (m/z 83 and 85). Chloroform standards (10–24,000 µg/L) were prepared with chloroform (99.9%) (02487-5ML, Sigma Aldrich, St. Louis, MO, USA) and GC-headspace grade methanol (414816-1L; Honeywell, Morris Plains, NJ, USA) and diluted in Milli-Q water. The calibration curve (0.01–24 µg/L) was made by adding 10 µL of the standard solution and 2 g of sodium chloride to 10 mL of Milli-Q water. The method was linear between 0.024–24 µg/L and the limit of detection (LOD) was 0.01 µg/L, while the limit of quantification was set to 0.024 µg/L. The SD was 6% at 0.024 µg/L and 3% at 0.2 µg/L. Chloroform in urine and water samples were analyzed at the Department of Chemical Work Environment, National Institute of Occupational Health, Oslo, Norway.

Creatinine concentration was also analyzed for each urine sample in an enzymic creatinine assay (Fossati et al. 1983) at the Department of Laboratory Medicine at Örebro University Hospital, Örebro, Sweden. This made it possible to compensate for individual differences in urine dilution, i.e. differences in water intake, in the determination of chloroform concentration.

### Statistical methods

Descriptive statistics were used for all data collected. The categorical baseline characteristics of the swimming pool workers were presented as percentages, and the continuous characteristics as arithmetic mean (AM), median, and range (min-max). Chloroform concentrations in the urine samples were corrected for creatinine concentrations.

The formula for creatinine correction:


$$\begin{array}{l} U-chloroform\;(\mu g/g\;creatinine)=\frac{U-chloroform\;(\mu g/L)}{U-creatinine(\mu g/g)} \end{array}$$

Chloroform concentrations in water, urine, and air were presented as AM, SD, median, geometric mean (GM), geometric standard deviation (GSD), and range. Using a Shapiro-Wilk test, it was determined that the chloroform concentrations in the urine samples had a skewed distribution, and the data were thus log-transformed before analysis.

Creatinine-corrected chloroform concentrations in urine were analyzed in a linear mixed model analysis to investigate the association with (i) time spent at the workplace when the workers submitted their urine samples, (ii) chloroform concentrations in personal air samples, (iii) degree of exposure based on whether the worker spent less than 50% or 50% or more of their working day near the swimming pools, and (iv) if the swimming pool workers executed any work tasks in the swimming pool water during the day of the investigation. The linear mixed model analysis was also executed with uncorrected chloroform concentrations in urine.

The formula for the linear mixed model analysis:


$$\begin{array}{l} Dependent\;variable\;(ln\;chloroform\;in\;urine)\;=\;\beta_{o}+\;\beta_{1}+\;ei \end{array}$$

ßo = intercept, a constant value that is the same for all participants.

Analysis 1: ß_1_ = time at work when submitting a urine sample (h); divided into groups: ≤2 (reference), >2–5, >5–10, and continuous.

Analysis 2: ß_1_ = chloroform in the air (µg/m^3^), divided into groups: ≤17.00 (reference), <17.01–28.00, >28.00 and continuous.

Analysis 3: ß_1_ = exposure time near swimming pools (%), divided into groups: <50 (reference), ≥50

Analysis 4: ß_1_ = work tasks in swimming pool water, divided into groups: no (reference), yes

ei = measurement error (residual)

The results of the linear mixed model were anti-log transformed after the analysis and were presented as odds ratios (OR) with a 95% confidence interval (CI). The strength of the association between chloroform concentrations in air and urine was calculated using the nonparametric Spearman’s rank-order correlation, where chloroform concentrations in the study participants’ fourth urine sample provided after >5–10 h at work and personal air chloroform concentrations from the same participants were used.

All analyses were made using IBM SPSS 27. *P*-values of ≤ 0.05 were considered statistically significant.

## Results

The study included five indoor adventure swimming pool facilities with a total of 41 study participants who had not worked at the swimming pool facilities in the last 48 h. During the day of the investigation, the participants were working a day- or evening shift with working hours starting between 05.45 and 16.00 and ending between 10.00 and 23.00. All participants were asked to provide up to four urine samples, and their personal exposure to air concentrations of chloroform was measured during the whole workday. Chloroform concentrations in swimming pool water were also measured. In addition, the study participants recorded their work tasks in a work diary and also completed a questionnaire. One work diary from one of the participants was missing and was thus not included in the linear mixed model analysis investigating the association of chloroform concentrations in urine with time spent near the swimming pools and executing work tasks in the swimming pool water.

### Demographics

All of the 41 adventure swimming pool workers included in the study answered the distributed questionnaire. However, internal data loss occurred as some questions (randomly) were left unanswered. The study participants consisted of more women (59%), and the mean age was 39 years. The majority were never smokers (61%), and they had worked for an average of 11 years at their current workplace. The swimming pool workers had a mean BMI of 26. The baseline characteristics are shown in Table 1.

**Table 1.** Baseline characteristics of the adventure swimming pool workers (*n* = 41).

Background variable		Swimming pool workers (%)
Gender	Male	17 (41)
	Female	24 (59)
Age (years)	AM ±SD	39 ± 13
	Median	38
	Min-max	19–68
Smoking habits	Never smoked	25 (61)
	Ex-smoker	11 (27)
	Smoker	5 (12)
Years at current workplace	AM ±SD	11 ± 10
	Median	10
	Min-max	0–35
BMI (kg/m^2^)	AM ±SD	26 ± 4
	Median	26
	Min-max	19–34

*n*, number of samples; AM, arithmetic mean; SD, standard deviation; BMI, body mass index.

### Chloroform concentrations in urine, air, and swimming pool water

For the urine analysis, the participants could provide up to four urine samples, and a total of 129 urine samples were collected. All urine samples were corrected for creatinine. Three subjects provided only one urine sample, 38 provided two samples, 29 submitted three samples, and 21 subjects provided four samples. The time at which the urine samples were submitted was different for each subject. To be able to relate urine concentrations of chloroform to time at work, the urine samples were divided into three groups based on the individual’s time at work before each urination. A total of 26 urine samples were submitted within 2 h of work, 54 samples were provided in the time range >2–5 h, and 49 samples were submitted >5 h after the start of the working day for each subject. The chloroform concentrations in the urine samples were shown to have a skewed distribution. The arithmetic mean chloroform concentrations in urine was 0.025 µg/g creatinine among the individuals with ≤2 h at work and 0.062 µg/g creatinine in the samples submitted by individuals with >2–5 working hours and 0.061 µg/g creatinine among the group with >5–10 working hours. The geometric mean chloroform concentrations in urine among the same groups was 0.009 µg/g creatinine in the group with the least number of hours at work, 0.023 µg/g creatinine in the middle group, and 0.026 µg/g creatinine in the group with the longest time at work. Sampling times for personal chloroform concentrations in air varied between 3.5 and 9.5 h. Chloroform concentrations in the air samples (*n* = 41) showed an arithmetic mean of 25 μg/m^3^ and a geometric mean of 11 μg/m^3^. The spread of these samples had an individual variation from 0.10 to 110 μg/m^3^. During the day of the investigation, water samples were collected in each swimming pool at the five different facilities (two measurements at one of the facilities) and analyzed for chloroform. A total of 36 samples were collected, and the arithmetic mean chloroform concentration in the different swimming pools was 6.6 µg/L and varied between 0.08 and 20.1 µg/L. The chloroform concentrations in urine, air, and swimming pool water are shown in Table 2. Chloroform concentrations in urine uncorrected for creatinine are shown in Supplementary Table S1.

**Table 2.** Chloroform concentrations in urine samples (corrected for creatinine) and air samples among the adventure swimming pool workers (*n* = 41) and chloroform concentrations in water samples from all swimming pools in the different facilities (*n* = 5).

		Chloroform						
	Time at work (h)	*n*	AM	SD	Median	GM	GSD	Min-max
Urine samples (µg/g creatinine)	≤2	26	0.025	0.040	0.011	0.009	4.6	0.00076–0.18
	>2–5	54	0.062	0.080	0.028	0.023	5.3	0.00081–0.35
	>5–10	49	0.061	0.067	0.031	0.026	4.9	0.00067–0.29
Total		129	0.054	0.070	0.024	0.020	5.2	0.00067–0.35
Air samples (μg/m^3^)								
Total		41	25	24	21	11	6.0	0.10–110
Water samples (µg/L)								
Total		36	6.6	5.7	7.3	2.3	5.6	0.08–20.1

*n*, number of samples; AM, arithmetic mean; SD, standard deviation; GM, geometric mean; GSD, geometric standard deviation.

The association of chloroform concentrations in urine and the time the subjects had spent at work prior to providing the urine samples were investigated in a linear mixed model analysis. Compared to the chloroform concentrations in the urine samples among the group with the shortest time at work (≤2 h), a significantly higher odds ratio of having a higher urinary chloroform concentration was found in the group with the second longest time spent at work (>2**–**5 h, OR 2.29, 95% CI 1.38–3.80) as well as in the group with the longest time at work (>5–10 h, OR 2.04, 95% CI 1.25–3.34). A linear mixed model was also used to determine the risk of having a higher urine concentration of chloroform if being in an exposure group of higher air concentration of chloroform. The group with the lowest air concentration of chloroform (≤17.00 µg/m^3^) was used as a reference for the others. The analysis showed a significantly higher odds ratio of having a higher chloroform concentration in urine in the group with the second highest chloroform concentrations in air (>17.00–28.00 µg/m^3^, OR 4.14, 95% CI 1.69–10.15) as well as in the group with the highest chloroform concentrations in air (>28.00 µg/m^3^, OR 9.23, 95% CI 3.68–23.13). Study participants who, according to their work diary, spent 50% or more of their working day near the swimming pools also had an increased risk of higher urinary concentrations of chloroform (OR 3.16, 95% CI 1.33–7.55) compared to workers with less time spent in the swimming pool area. Ten participants executed work tasks in the swimming pool water. They spent approximately 15–70% of their workday in the water, but they were not found to have an increased risk of higher chloroform concentrations in urine in comparison to workers who did not spend working time in the water (OR 0.82, 95% CI 0.27–2.45). The results from the linear mixed model analyses are given in Table 3. Chloroform concentrations in the urine associated with chloroform concentrations in the air have also been analyzed as continuous variables in a linear mixed model (β = 0.038; 95% CI 0.023–0.053, *P* < 0.001) in combination with hours at work at each urine sampling as a continuous variable (β = 0.000026; 95% CI 0.000003–0.0000048, *P* = 0.025). The results from the linear mixed model analyses with uncorrected chloroform concentrations are shown in Supplementary Tables S2 and S3. A Spearman’s rank correlation between the chloroform concentration (corrected for creatinine) in the fourth urine sample provided after >5–10 h at work and the corresponding air samples (*n* = 21) were found to be 0.696 with a *P*-value of < 0.001. (Spearman’s rank correlation with uncorrected chloroform concentrations in urine: 0.754, *P*-value < 0.001).

**Table 3.** Linear mixed model analysis of chloroform concentrations in urine samples (corrected for creatinine) in different groups based on time at work before providing the urine sample, chloroform concentrations in air, proportion of time spent near the swimming pools during the workday and if the workers executed works tasks in the swimming pool water. Bold font indicates statistical significance (*P* < 0.05).

		*n*	OR^b^	95% CI
Time at work (h)	≤2^a^	26	1	
	>2**–**5	49	**2.29**	**1.38–3.80**
	>5**–**10	54	**2.04**	**1.25–3.34**
Chloroform in air (µg/m^3^)	≤17.00^a^	46	1	
	>17.00**–**28.00	41	**4.14**	**1.69–10.15**
	>28.00	42	**9.23**	**3.68–23.13**
Time near the swimming pools (%)	<50^a^	49	1	
	≥50	76	**3.16**	**1.33–7.55**
Work tasks in swimming pool water	No^a^	96	1	
	Yes	29	0.82	0.27**–**2.45

Bold font indicates statistical significance (*P* < 0.05).

*n*, number of samples; OR, odds ratio; CI, confidence interval.

^a^reference,

^b^anti-log transformed.

## Discussion

The purpose of this study was to investigate if chloroform concentrations in air and water affect the chloroform concentration in urine samples of exposed swimming pool workers. Other THMs are also known to be present in swimming pool air, but chloroform has been found to be the dominant THM in this type of environment (Westerlund et al. 2015). Chloroform can either be metabolized in the liver or excreted unmetabolized through expired air and urine (Agency for Toxic Substances and Disease Registry 1997; Gemma et al. 2003). An earlier study found a correlation between long-term exposure to chloroform and bladder cancer (Costet et al. 2011), an increase in urine chloroform concentration could be a part of this correlation.

The chloroform concentrations in the urine samples were shown to have a skewed distribution. Comparison of the geometric mean chloroform concentrations in urine samples after ≤2 h at work (0.009 µg/g creatinine) and after >5–10 h at work (0.026 µg/g creatinine) show an increase of chloroform levels during the work shift. This is also seen in the linear mixed model analysis, which shows a significant relationship between longer time at work before providing the urine sample and an increased risk of higher urinary levels of chloroform (β = 0.000026; 95% CI 0.000003–0.0000048, *P* = 0.025). This is consistent with an earlier study that investigated urine concentrations of THMs in workers and visitors before and after exposure to an indoor swimming pool, which showed increased THM concentrations in urine 16 h after exposure (Cammann and Hubner 1995). However, the chloroform concentrations in the urine samples do not increase as much later in the work day compared to concentrations in earlier samples (time at work >2–5 h: OR 2.29, 95% CI: 1.38–3.80 versus time at work >5–10 h: OR 2.04, 95% CI: 1.25–3.34). This may be due to the time during the work shift the workers worked in the swimming pool but it might also be explained by individual and diurnal variations in the metabolizing enzymes in the cytochrome P450, known to metabolize chloroform in the human liver (Snawder and Lipscomb 2000; Gemma et al. 2003; Košir et al. 2013). Gängler et al. (2018) studied diurnal variability in the CYP2E1, a member of the P450 enzyme family, in association with THM exposure during cleaning and measurement of THM concentrations in urine. They found the CYP2E1 to be more active during daytime, which meant lower THM levels in urine during the day compared to samples submitted in the evening. Thus, even our results are considered to be influenced by the variation in this enzyme system.

The use of air samples from the breathing zone of the subjects instead of stationary air sampling, which is used in many other swimming pool studies, made it possible to measure individual differences in exposure. Thus, in turn made it possible to analyze the effect of individual exposure and urine chloroform concentration. The result of measured chloroform concentrations in the ambient air of the swimming pool facilities, with a geometric mean of 11 μg/m^3^ and a maximum of 110 μg/m^3^, is well within the Swedish occupational exposure limit of 10,000 μg/m^3^ set by the SWEA (Swedish Work Environment Authority 2018). The maximum of 110 μg/m^3^ in our results is almost 100 times lower than the limit of 10,000 μg/m^3^. Earlier studies have measured chloroform concentrations of 21–340 μg/m^3^ in the ambient air of indoor swimming pools in Italy and Spain (Aggazzotti et al. 1998; Caro and Gallego 2008; Aprea et al. 2010; Fantuzzi *et al.*, 2001). Personal breathing zone results found in this study are within the other studies’ concentration ranges. The highest concentration of chloroform in swimming pool water found in this study was 20.1 µg/L. There is no limit value for chloroform in swimming pool water; however, the concentrations were found to be lower than the recommended upper limit of 100 µg/L for THMs in swimming pool water (Public Health Agency of Sweden 2021).

The result of the linear mixed model regression analysis showed a significant association between chloroform concentrations in urine and air concentrations as continuous variables (β = 0.038; 95% CI 0.023–0.053, *P* < 0.001) with an increased odds ratio of having higher urine chloroform concentration in the more exposed groups (≤17.00 µg/m^3^, OR 1; >17.00–28.00 µg/m^3^, OR 4.14, 95% CI 1.69–10.15 and >28.00 µg/m^3^, OR 9.23, 95% CI 3.68–23.13). This shows that increasing exposure to chloroform in air increases concentrations in urine of indoor swimming pool workers. The Spearman’s rank correlation showed a correlation of 0.696 (*P* < 0.001), which further shows an association of air and urine concentration of chloroform.

THM concentrations in air are known to be higher closer to swimming pools compared to concentrations found in adjacent premises (Fantuzzi et al. 2010; Nitter et al. 2019). The swimming pool workers included in the study had varying work tasks during the day of the investigation. They executed tasks such as administrative work outside the pool area, lifeguard duties inside the pool area and some of them also taught swimming school in the swimming pool water. The differences in work tasks during the day of the investigation were recorded by each participant in a work diary. Spending half or the majority of the workday (≥50%) near a swimming pool was found to be significantly associated with an increased risk of higher levels of chloroform in the urine compared to workers who spent less time in the pool area (OR 3.16, 95% CI 1.33–7.55).

The major pathway of THM uptake in a swimming pool environment is inhalation, though they can also enter the body through dermal absorption (Erdinger et al. 2004). This could mean that working in swimming pool water results in exposure to chloroform via both the skin and respiratory tract and thus higher levels in the urine compared to those with respiratory exposure alone. However, the employees executing work tasks in swimming pool water in this study could not be associated with a higher risk of more chloroform in urine than employees only working on land (OR 0.82, 95% CI 0.27–2.45). The air samplers could not be worn while the subjects were in the water, which could impact the total concentration in the air samples. While the participants were in the water, the samplers were placed next to the pool and continued to collect chloroform in order to limit the effect on the results. The air closest to the water surface has the highest chloroform concentrations (Erdinger et al. 2004), which means that the air sample results could be lower than the true exposure.

A limitation of the study is the lack of a urine sample before the exposure. The first sample of each subject was collected after different amounts of time at the swimming pool facilities. A sample from each subject before entering the swimming pool area would have been a better reference in the comparison of chloroform concentration in the different urine samples during their work shift. The inclusion criteria used in this study, i.e. that the subjects had to be free from work the previous 48 h, partly mitigates this, as an earlier study has shown that urine THM concentrations are normalized within 15 h (Cammann and Hubner 1995). Sweden also uses low amounts of chlorine-based disinfection in its water supply (Swedish Food Agency 2001). Due to this, exposure to THMs from drinking water and showers while away from the swimming pool is probably very low and not likely to influence our results. An additional source for THM exposure among swimming pool workers is cleaning chemicals. Cleaning chemicals can contain chlorine, which in turn has been seen to increase the levels of THM concentration in urine (Charisiadis et al. 2014; Ioannou et al. 2019). The use of cleaning chemicals, and thus additional source of THM exposure, in the swimming pool facilities included in this study was not registered.

The uncertainty of the health risks of chloroform and other THMs makes the result of this study important, especially as the air and water concentrations in this study were well within the Swedish regulations and recommendations. An epidemiological study from Spain showed an increased risk of bladder cancer with higher exposure to THMs through ingestion, dermal uptake, and inhalation. It also showed an increased risk of bladder cancer with more swimming pool exposure (Villanueva et al. 2007). THM levels in drinking water in the European Union have been studied during the years 2005–2018, and a mean THM level of 11.7 µg/L has been estimated as a likely burden of bladder cancer (Evlampidou et al. 2020). Chloroform concentrations in air (0.10–110 µg/m^3^) and swimming pool water (0.08–20.1 µg/L) measured in this study need to be considered as an additional THM exposure for swimming pool workers and may thus affect bladder cancer burden in this population. In their guidelines on swimming pools, the Public Health Agency of Sweden states that the increased risk of cancer associated with THM exposure in swimming pools is negligible but also says that the assessment is based on assumptions and uncertainties (Public Health Agency of Sweden 2021).

This increases the need for further studies of the health risks of chloroform, in order to be able to assess the current regulations. More studies on the levels of THMs that visitors and workers of indoor swimming pools are exposed to, and the effect this long-term exposure has on them, are needed. Further, epidemiological studies on swimming pool workers and cancer would be needed to assess the effect of THMs and other DBPs in this environment.

## Conclusions

This study found an accumulation of chloroform concentrations in urine during a workday and a correlation between breathing zone and urine concentrations of chloroform among workers in Swedish indoor swimming pools. Our results indicate a further need for studies on the long-term effects of chloroform exposure.

## Supplementary Material

wxad035_suppl_Supplementary_Material_1

wxad035_suppl_Supplementary_Material_2

wxad035_suppl_Supplementary_Material_3

## Data Availability

The data underlying this article cannot be shared publicly due to the privacy of individuals that participated in the study. The data will be shared on reasonable request to the corresponding author.

## Notes

**Clinical significance:** The clinical significance of this paper is the finding that occupational chloroform exposure in air in indoor swimming facilities has a statistically significant relation to chloroform concentrations in urine, which can be used to further elucidate chemical exposure among swimming pool workers and protect workers’ health.
