# Supplementary material for: Chloroform exposure in air and water in Swedish indoor swimming pools—urine as a biomarker of occupational exposure
Source: Ann Work Expo Health. 2023 Jun 20;67(7):876–85. doi: 10.1093/annweh/wxad035 (PMC10410492; doi:10.1093/annweh/wxad035)
Supplement: wxad035_suppl_Supplementary_Material_1 [file wxad035_suppl_supplementary_material_1.docx]

# Supplementary material 1

**Chloroform exposure in air and water in Swedish indoor swimming pools - urine as a biomarker of occupational exposure**

Oskar Ragnebro^1#^, BSc, Kristin Helmersmo^2#^, MSc, Louise Fornander^3^, PhD, Raymond Olsen^2^, PhD, Ing-Liss Bryngelsson^3^, BSc, Pål Graff^2^, PhD and Jessica Westerlund^3^, PhD

^1^School of Medical Sciences, Örebro University, Örebro, Sweden

^2^National Institute of Occupational Health (STAMI), Oslo, Norway

^3^Department of Occupational and Environmental Medicine, Faculty of Medicine and Health, Örebro University, Örebro, Sweden

# These authors contributed equally.

**ATD method settings.**

| Tube Temperature | °C | 250 |
| --- | --- | --- |
| Transferline Temperature | °C | 205 |
| Valve Temperature | °C | 205 |
| Trap Low Temperature | °C | 5 |
| Trap High Temperature | °C | 250 |
| Trap Rate | °C/s | 40 |
| Desorb Tube Time | min | 5 |
| Trap hold | min | 4 |
| Desorb Trap Time | min | 1 |
| Desorb Flow Rate | ml/min | 50 |
| Outlet split | ml/min | 10 |

**GC temperature program.**

| Rate | Temperature | Hold time | Run time |
| --- | --- | --- | --- |
| °C/min | °C | min | min |
|  | 35 | 1.00 | 1.00 |
| 5 | 125 | 15.00 | 34.00 |
