# Supplementary material for: Chloroform exposure in air and water in Swedish indoor swimming pools—urine as a biomarker of occupational exposure
Source: Ann Work Expo Health. 2023 Jun 20;67(7):876–85. doi: 10.1093/annweh/wxad035 (PMC10410492; doi:10.1093/annweh/wxad035)
Supplement: wxad035_suppl_Supplementary_Material_2 [file wxad035_suppl_supplementary_material_2.docx]

# Supplementary material 2

**Chloroform exposure in air and water in Swedish indoor swimming pools - urine as a biomarker of occupational exposure**

Oskar Ragnebro^1#^, BSc, Kristin Helmersmo^2#^, MSc, Louise Fornander^3^, PhD, Raymond Olsen^2^, PhD, Ing-Liss Bryngelsson^3^, BSc, Pål Graff^2^, PhD and Jessica Westerlund^3^, PhD

^1^School of Medical Sciences, Örebro University, Örebro, Sweden

^2^National Institute of Occupational Health (STAMI), Oslo, Norway

^3^Department of Occupational and Environmental Medicine, Faculty of Medicine and Health, Örebro University, Örebro, Sweden

# These authors contributed equally.

**ITEX direct head space method settings.**

| Agitator Incubation Temperature | °C | 60 |
| --- | --- | --- |
| Agitator Incubation Time | min | 5 |
| Agitator Speed | rpm | 500 |
| Syringe Temperature | °C | 60 |
| Trap Purge Time | s | 60 |
| Trap Extraction Temperature | °C | 30 |
| Sample Volume | µL | 700 |
| Sample Vial Penetration Depth | mm | 12 |
| Extraction Aspirate Flow Rate | µL/s | 30 |
| Extraction Dispense Flow Rate | µL/s | 200 |
| Extraction Strokes | number | 20 |
| Extraction Volume | µL | 1000 |
| Extraction Pullup Delay | s | 5 |
| Water Removal Trap Temperature | °C | 50 |
| Water Removal Purge Time | s | 120 |
| Desorption Flow Rate | µL/s | 20 |
| Desorption Temperature | °C | 170 |
| Trap Post Cleaning Time | s | 600 |
| Trap Post Cleaning Temperature | °C | 210 |
| Water Removal Trap Temperature | °C | 50 |
| Water Removal Purge Time | s | 120 |

**GC temperature program.**

| Rate | Temperature | Hold time | Run time |
| --- | --- | --- | --- |
| °C/min | °C | min | min |
|  | 30 | 10 | 10 |
| 8 | 155 | 0 | 25.625 |
| 50 | 200 | 5 | 31.525 |

**GC carrier gas flow rate program.**

| Flow Rate | Hold time | Run time |
| --- | --- | --- |
| mL/min | min | min |
| 0.8 | 2 | 2 |
| 1.5 |  | 31.525 |
