# Supplementary material for: Chloroform exposure in air and water in Swedish indoor swimming pools—urine as a biomarker of occupational exposure
Source: Ann Work Expo Health. 2023 Jun 20;67(7):876–85. doi: 10.1093/annweh/wxad035 (PMC10410492; doi:10.1093/annweh/wxad035)
Supplement: wxad035_suppl_Supplementary_Material_3 [file wxad035_suppl_supplementary_material_3.docx]

# Supplementary material 3

**Chloroform exposure in air and water in Swedish indoor swimming pools - urine as a biomarker of occupational exposure**

Oskar Ragnebro^1#^, BSc, Kristin Helmersmo^2#^, MSc, Louise Fornander^3^, PhD, Raymond Olsen^2^, PhD, Ing-Liss Bryngelsson^3^, BSc, Pål Graff^2^, PhD and Jessica Westerlund^3^, PhD

^1^School of Medical Sciences, Örebro University, Örebro, Sweden

^2^National Institute of Occupational Health (STAMI), Oslo, Norway

^3^Department of Occupational and Environmental Medicine, Faculty of Medicine and Health, Örebro University, Örebro, Sweden

# These authors contributed equally.

**Table S1.** Unadjusted urinary chloroform concentrations among the adventure swimming pool workers (*n* = 41) divided into groups based on the individual’s time at work before each urination.

|  |  | Chloroform | | | | | | |
| --- | --- | --- | --- | --- | --- | --- | --- | --- |
|  | Time at work (h) | *n* | AM | SD | Median | GM | GSD | Min-max |
| Urine samples (µg/L) | ≤2 | 26 | 0.022 | 0.034 | 0.0071 | 0.010 | 3.8 | 0.00071–0.16 |
|  | >2–5 | 54 | 0.035 | 0.059 | 0.021 | 0.016 | 3.7 | 0.00071–0.34 |
|  | >5–10 | 49 | 0.028 | 0.021 | 0.029 | 0.015 | 4.6 | 0.00032–0.083 |
| Total |  | 129 | 0.030 | 0.043 | 0.022 | 0.014 | 4.0 | 0.00032–0.34 |

*Abbreviations:* n: number of samples; AM: arithmetic mean; SD: standard deviation; GM: geometric mean; GSD: geometric standard deviation

**Table S2.** Linear mixed model analysis of unadjusted urinary chloroform concentrations (µg/L) from swimming pool workers divided into different groups based on time at work before providing the urine sample, chloroform concentrations in air, proportion of time spent near the swimming pools during the workday and if the workers executed works tasks in the swimming pool water. Bold font indicates statistical significance (p < 0.05).

|  |  | *n* | OR^b^ | 95% CI |
| --- | --- | --- | --- | --- |
| Time at work (h) | ≤2^a^ | 26 | 1 |  |
|  | >2**–**5 | 49 | **1.89** | **1.23–2.90** |
|  | >5**–**10 | 54 | 1.35 | 0.89–2.04 |
| Chloroform in air (µg/m^3^) | ≤17.00^a^ | 46 | 1 |  |
|  | >17.00**–**28.00 | 41 | **4.85** | **2.46–9.55** |
|  | >28.00 | 42 | **7.02** | **3.51–14.01** |
| Time near the swimming pools (%) | <50^a^ | 49 | 1 |  |
|  | ≥50 | 76 | **2.47** | **1.19–5.14** |
| Work tasks in swimming pool water | No^a^ | 96 | 1 |  |
|  | Yes | 29 | 1.21 | 0.49–3.02 |

^a^reference, ^b^anti-log transformed

*Abbreviations:* OR: odds ratio; CI: confidence interval

**Table S3.** Linear mixed model analysis of unadjusted urinary chloroform concentrations (µg/L) (n=129) and chloroform concentrations in air (n=41) as well as exposure time.

| Parameter | Estimate^a^ | p-value | 95% CI |
| --- | --- | --- | --- |
| Chloroform in air (µg/m^3^) | 0.031 | < 0.001 | 0.019–0.044 |
| Exposure time (h) | 0.000012 | 0.220 | -0.000007-0.000031 |

^a^log transformed

*Abbreviations:* n: number of samples; CI: confidence interval
